# Recent Advances in Hybrid Brain-Computer Interface Systems: A Technological and Quantitative Review

**DOI:** 10.32598/bcn.9.5.373

**Published:** 2018-09-01

**Authors:** Sahar Sadeghi, Ali Maleki

**Affiliations:** 1. Department of Biomedical Engineering, Faculty of New Sciences and Technologies, Semnan University, Semnan, Iran.

**Keywords:** Brain-Computer Interfaces (BCI), BCI control signal, Human-machine interface biosignal, Simultaneous and sequential HBCI

## Abstract

Brain-Computer Interface (BCI) is a system that enables users to transmit commands to the computer using their brain activity recorded by electroencephalography. In a Hybrid Brain-Computer Interface (HBCI), a BCI control signal combines with one or more BCI control signals or with Human-Machine Interface (HMI) biosignals to increase classification accuracy, boost system speed, and improve user’s satisfaction. HBCI systems are categorized according to the type of combined signals and the combination technique (simultaneous or sequential). They have been used in several applications such as cursor control, target selection, and spellers. Increasing the number of articles published in this field indicates the significance of these systems. In this paper, different HBCI combinations, their important features, and potential applications are discussed. In most cases, the combination of a BCI control signal with a HMI biosignal yields higher information transfer rate than two BCI control signals.

## Highlights

- In the hybrid brain-computer interface system, a brain-computer interface signal combines with other brain-computer interface signals or with human-machine interface biosignals.
- Sequential combination reduces errors and simultaneous combination increases the information transfer rate.
- Combination of brain-computer interface signal with human-machine interface biosignal yields higher information transfer rate than two brain-computer interface signals.
- The EEG+Eye Tracker and SSVEP+ERD have achieved highest and lowest information transfer rate, respectively.
- The EEG+NIRS has the lowest accuracy in comparison with other hybrid brain-computer interface combinations.

## Plain Language Summary

Individuals with ALS, brainstem stroke, spinal cord injury and patients with numerous diseases may lose most or all voluntary muscle control so that sometimes they will not be able to speak well. The Brain-Computer Interface (BCI) system provides an alternative pathway between their brain and the system and enables these people to control their environment. It has been used in several applications such as cursor control, target selection, and spellers. Since a BCI system based on one method may not work on all subjects, the hybrid Brain-Computer Interface (HBCI) system has been introduced. In a HBCI system, a BCI control signal combines with one or more BCI control signals or with Human-Machine Interface (HMI) biosignals. Increasing the number of articles published in this field indicates the significance of these systems.

In this paper, different HBCI combinations, their important features, and potential applications are discussed. Classification accuracy and Information Transfer Rate (ITR) are two important parameters to evaluate a BCI system. It is a trade-off between these two parameters; as one increases, the other one decreases and vice versa. Therefore, concerning the ultimate goal and depending on the application, the appropriate combination type should be determined. This paper makes it easier to choose the optimal combination, by illustrating the correct location of the accuracy and the ITR corresponding to each combination. Results of this study show that in most cases, the combination of a BCI signal with a HMI biosignal yields higher ITR than two BCI control signals. The highest and lowest ITR were achieved using EEG+Eye Tracker and SSVEP+ERD, respectively. The combination of EEG signal and NIRS had also the lowest accuracy, while the accuracy values of other hybrid systems did not differ much from each other and they were located within the range of 70% to 100%.

## 1. Introduction

**Figure 1.**
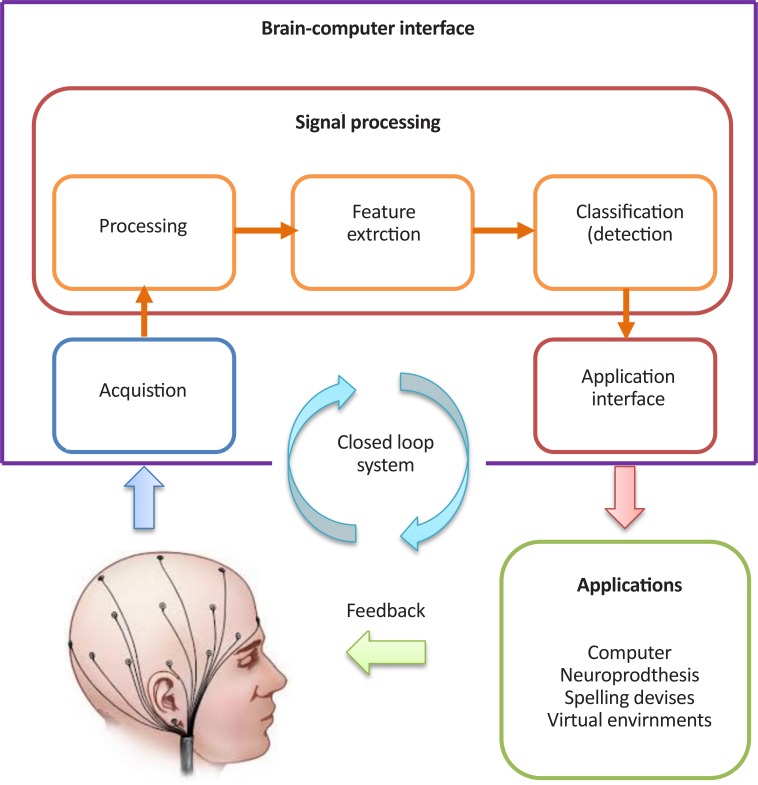
Block diagram of a BCI system

A Brain-Computer Interface (BCI) system provides a non-muscular communication channel by creating a direct path between brain and computer aimed at communicating with environment for people suffering from severe paralysis, muscular atrophy, amyotrophic lateral sclerosis or brainstem stroke. BCI consists of sensors and signal processing tools that directly convert brain activity into commands or messages (Müller-Putz et al., 2012; Guger, Allison, & Müller Putz, 2015). The block diagram of the BCI system is illustrated in Figure 1. Brain activity is measurable using several approaches such as electroencephalography, magneto-encephalography, functional Magnetic Resonance Imaging (fMRI), Electrocorticography (ECoG), and Near-Infrared Spectroscopy (NIRS) (Amiri, Rabbi, Azinfar, & Fazel-Rezai 2013).

Electroencephalogram (EEG) signal is often considered as the input in most BCI systems. EEG electrodes are placed on the scalp and special devices record the electric field of neural activity. Six brain rhythms can be distinguished in EEG signal based on the differences in frequency ranges: Delta (1–4 Hz), theta (4–7 Hz), alpha (8–12 Hz), mu (8–13 Hz), beta (12–30 Hz), and gamma (25–100 Hz). Delta rhythm includes all rhythms below 3.5 Hz and is generated during deep sleep, by children or the people with brain disorders.

Theta rhythm is further acquired from temporal and parietal areas and it is visible in children and some adults at the time of stress, disappointment, and heartbreak. Alpha rhythm occurs in awake and eyes closed relax condition. It is significantly recorded from the occipital lobes, but it can also be acquired from parietal and frontal regions. This rhythm completely disappears during sleep and when the subject is attracted to a particular mental activity in the waking state, it is replaced with asynchronous waves with higher frequency and lower amplitude. Beta rhythm is mostly acquired from parietal and frontal areas.

It takes place at frequencies as high as 50 Hz in strong brain activities. It is divided into Beta I and Beta II. Beta I, with the frequency of about twice as Alpha rhythm, and influenced by similar mental activities affecting the alpha rhythm. Beta II appears in the central nervous system during intense activities and in the time of stress. The alpha activity, which is recorded from the sensory-motor areas, is called mu activity. Gamma rhythm acquired from somatosensory cortex is involved in high-level tasks such as cognitive functions and it is important for learning, memory, and data processing (Amiri et al., 2013; Bharne & Kapgate, 2015).

In general, BCI systems are categorized based on the brain activity patterns into four different types: P300 component of event-related potential, Steady-State Visual Evoked Potential (SSVEP), SCP, and Event-Related Synchronization (ERS)/Event-Related Desynchronization (ERD).

P300 is an Event-Related desynchronization Potential (ERP) that appears approximately 300 ms after a visual, auditory or tactile stimulation. Since P300-based BCI systems are vulnerable against noise, they require averaging of ERP responses from several stimuli. This reduces the speed and Information Transfer Rate (ITR). These systems also need less training and they have high validity among users and patients (Fazel-Rezai & Ahmad, 2011; Fazel-Rezai et al., 2012; Wolpaw & Wolpaw, 2012).

Visual Evoked Potentials (VEPs) are brain oscillations that occur after receiving a visual stimulation. Steady-State Visual Evoked Potential (SSVEP) is a kind of VEP that occurs in response to stimulus with frequencies higher than 6 Hz. Although higher stimulation frequencies reduce fatigue and discomfort, the recognition of the signal is challenging. In general, SSVEP-based BCI systems have many advantages such as better classification accuracy, higher ITR, and fewer numbers of required electrodes, compared to other methods such as P300. These systems do not need training and if necessary, the required time for training is very short. Although SSVEP-based systems are faster than systems based on P300, they have shortcomings such as inappropriateness for patients with epilepsy, requirement of precise control of eye muscles, and the need for high-speed hardware (Guger et al., 2012; Guger et al., 2015).

Slow-Cortical Potentials (SCP) are negative slow potential changes in EEG signals acquired in an imagined or actual movement from sensory-motor cortex of the brain (Allison et al., 2012; Faller, 2012). These potentials belong to the part of EEG signals with frequencies less than 1 Hz and are a reflection of cortical polarization. The employment of these potentials is limited due to reasons such as long duration training time, high error risk, and poor dimensional control.

Mu and Beta rhythms, both recorded from the sensory-motor cortex, are caused by sensory stimulation or motor behavior. These rhythms include two types of amplitude fluctuations named ERD and ERS. A voluntary movement causes a limited asynchronicity in the lower bands of Mu and Beta that is called ERD and it takes place about two seconds before starting the movement. In fact, the decrease in neuronal synchronization reduces power in specific frequency bands and eventually reduces the signal amplitude. After a voluntary movement, with an increase in synchronization of neurons, the power is increased in brain rhythms and reaches its maximum level, 600 ms after the movement that is called ERS. The Motor Imagery (MI) is also a way to change in ERD/ERS favored in BCI applications. This method requires more training and may not applicable on some subjects (Pfurtscheller & Da Silva, 1999).

From another perspective, BCI systems are categorized into synchronous and asynchronous. In a synchronous system, the extraction and processing of signal features are prescheduled. It is based on the protocol that defines the starting and ending of each operation with specified duration. In an asynchronous system, called automated system, feature extraction and processing do not necessarily follow a fixed schedule.

## 2. Methods

### 2.1. Hybrid brain- computer interface systems

Since a BCI system based on one method may not work on all subjects, the Hybrid BCI (HBCI) system is introduced and this area is of interest to many researchers over time.

The current study aimed at reviewing and analyzing the current state-of-the-art HBCI studies. In this review, articles were sought from the Google scholar database. Inclusion criteria were journal articles written in English from 2010 to December 2016. Other publication forms (eg, books, proceeding papers, master’s and doctoral dissertations, unpublished working papers, newspapers, etc.) were not included. Keywords used in search engines were “Hybrid” AND “Brain computer interface”, “Hybrid” AND “Brain machine interface”, “Brain computer interface” AND “Electroencephalography”, “Brain computer interface” AND “Electrooculography”, “Brain computer interface” AND “Electromyography”, “Brain computer interface” AND “Near-infrared spectroscopy”, “Brain computer interface” AND “evoked potential” OR “Brain computer interface” AND “Steady-state somatosensory evoked potential”.

**Figure 2.**
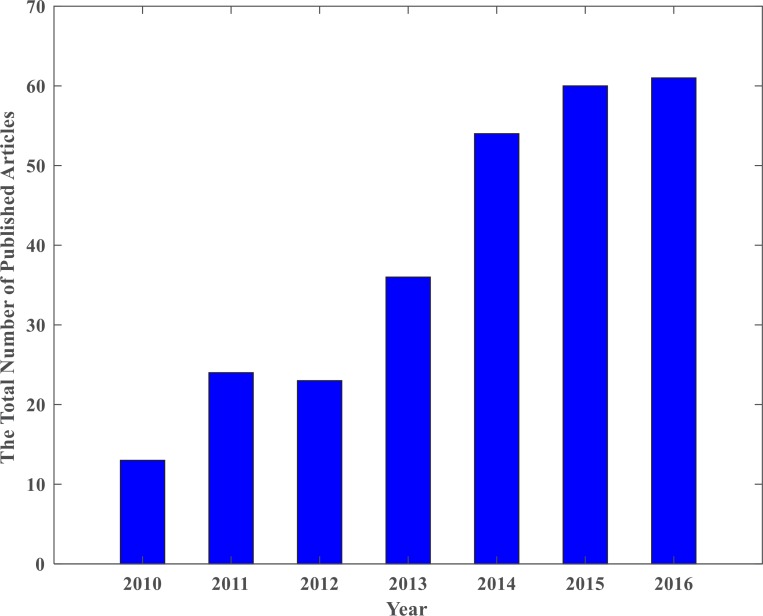
The Annual publication of articles based on Google Scholar database in the area of HBCI systems

After conducting the keyword search, some papers were found more than one time with different keywords. Therefore, duplicates were excluded. Figure 2 shows the total number of articles published in different years based on Google Scholar database. There were 13 articles in 2010 and this number significantly rose in the following years. A total number of 60 and 61 articles were published in this context in 2015 and 2016, respectively. Increasing the number of articles published in the realm of HBCI indicates the high efficiency hybrid brain-computer interface of these systems.

**Figure 3.**
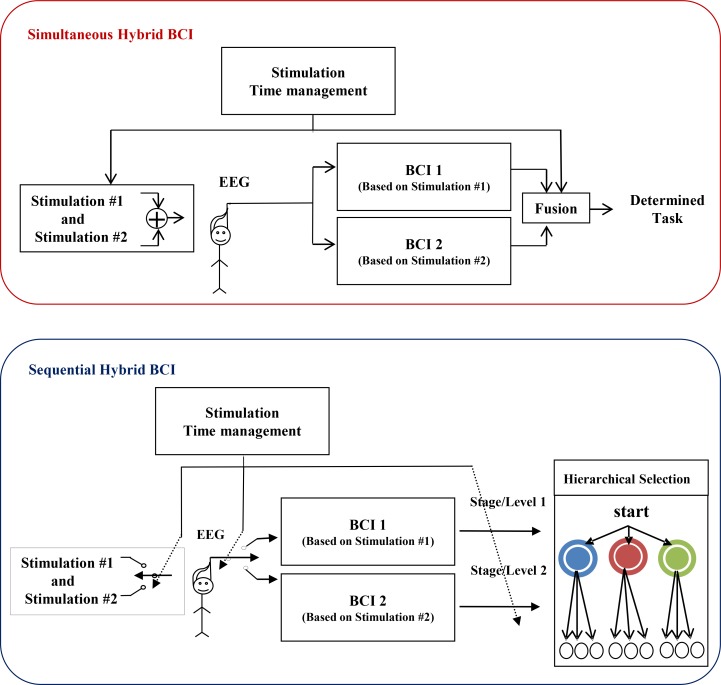
The types of combinations in HBCI systems Upper figure: Simultaneous combination, Lower figure: Sequential combination

In a HBCI, a BCI control signal combines with one or more BCI control signals or with Human Machine Interface (HMI) biosignals. HBCI systems are categorized according to the type of signals combined and the combination technique (Simultaneous/sequential). In simultaneous combination, the systems work concurrently with each other, while in sequential combination they act as time-sharing. In a sequential combination, the target is selected among several options by the first system and the second system does the process on the choice. A comprehensive block diagram with different modes of system operation is presented in Figure 3. This Figure completely describes the concept of system operation in both modes; simultaneous and sequential. The timing of stimulation in operation modes is depicted in this Figure.

In general, the most important goals of combining signals in HBCI systems are to increase the classification accuracy, enhancing system speed, improving user satisfaction, and overcoming the disadvantages of BCI systems. In contrast, most of these hybrid systems are associated with greater complexity.

### 2.2. Types of HBCI Systems

**Figure 4.**
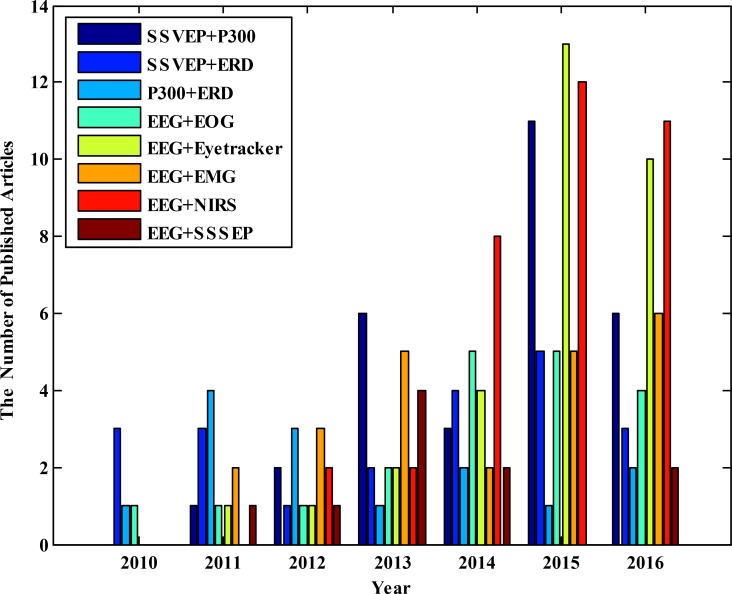
The annual publication of articles based on the type of HBCI combination obtained from Google Scholar database

To date, different combinations are employed in HBCI systems. Figure 4 shows the number of articles published in different years based on the type of combination, obtained from Google Scholar database. This Figure indicates that in the early years of the employment of HBCI systems, the combination of BCI control signals was used in various studies. Over the time, the combination of BCI control signals with HMI biosignals was also considered. Figure 4 shows the gradual increase in the use of Electromyogram (EMG), Electrooculogram (EOG), and Steady-State Somatosensory Evoked Potentials (SSSEP) in HBCI systems over the time. However, the use of NIRS and eye tracker increased dramatically, especially in recent years. A summary of studies in the field of HBCI systems with an emphasis on the specific characteristics of each study is noted in Table 1. In the following, with the introduction of a variety of signal combinations in HBCI systems, methods and results of different studies are investigated.

**Table 1.** Comparison of the specific characteristics of The Studies in the field of HBCI

**Paper**	**Combined Control Signals**	**Combination Type**	**Classification**	**Application**	**Result**
Yin et al. (2014)	P300+SSVEP	Sequential	FLDA, BLDA	Object control	Fast and accurate detection of the control state of subject
Wang et al. (2015)	P300+SSVEP	Sequential	SWLDA	Speller	Increase the classification accuracy
Bharne et al. (2015)	P300+SSVEP	Simultaneous	SWLDA	Speller	Reduce errors+increase the classification accuracy and ITR
Panicker et al. (2011)	P300+SSVEP	Simultaneous	SWLDA, CCA	Speller	Increase the classification speed
Edlinger et al. (2011)	P300+SSVEP	Simultaneous	BLDA, CCA	Target selection	Increase the classification speed
Yin et al. (2013)	P300+SSVEP	Sequential	LDA	Object control	Increase the classification speed
Xu et al. (2013)	P300+SSVEP	Simultaneous	SVM-FLDA	Curser movement	Inappropriate speed and ignoring the control state of subject due to system synchronization
Xu et al. (2013)	P300+SSVEP	Sequential	Kernel FDA, SVM	Object control	Increase the classification accuracy
Xu et al. (2013)	P300+SSVEP	Sequential	SVM	Object control	Increase the classification accuracy
Xu et al. (2013)	P300+SSVEP	Simultaneous	LDA, SWLDA	Speller	Increase the classification accuracy and ITR
Xu et al. (2013)	P300+SSVEP	Sequential	SVM, LDA	Speller	Increase the ITR
Allison et al. (2010)	SSVEP+ERD	Simultaneous	FLDA	Curser movement	Increase the classification accuracy
Savić et al. (2011)	SSVEP+ERD	Sequential	LDA	Orthotics control	Decrease the positive error rate
Brunner et al. (2011)	SSVEP+ERD	Simultaneous	LDA	Curser movement	Increase the classification accuracy and ITR
Pfurtscheller et al. (2010)	SSVEP+ERD	Sequential	-	Neural prosthesis control	Reduce the time spent
Allison et al. (2012)	SSVEP+ERD	Simultaneous	LDA	Curser movement	Continuous and simultaneous movement in two dimensions
Li et al. (2013)	SSVEP+ERD	Simultaneous	LDA	Wheelchair control	Simultaneous control of direction and speed
Li et al. (2014)	SSVEP+ERD	Simultaneous	SVM	Wheelchair control	Simultaneous set of direction and speed with spend the least possible time and high classification accuracy
Cao et al. (2014)	SSVEP+ERD	Simultaneous	RBF-SVM	Wheelchair control	Realization of eight control command
Cao et al. (2014)	SSVEP+ERD	Simultaneous	SVM	Object control	Achieve optimal performance
Rebsamen et al. (2008)	P300+ERD	Sequential	-	Wheelchair control	To determine and fulfill the stop command
Long et al. (2012a)	P300+ERD	Simultaneous	LDA	Wheelchair control	Direction and speed control
Finke et al. (2011)	P300+ERD	Sequential	FDA	Robot control	Providing movement in various dimensions
Su et al. (2011)	P300+ERD	Simultaneous	SVM, FLDA	Object control	Realization of more complex tasks
Riechmann et al. (2011)	P300+ERD	Sequential	LDA	Robot control	Robot control
Riechmann et al. (2011)	P300+ERD	Sequential	LDA	Speller	Increase the classification accuracy and ITR
Riechmann et al. (2011)	P300+ERD	Sequential	LDA	Speller	Increase the classification accuracy and ITR
Usakli et al. (2009)	EEG+EOG	Sequential	-	Robot control	Affective robot control
Riccio et al. (2015)	EEG+EOG	Simultaneous	SVM	Prosthesis control	Increase the classification accuracy
Riccio et al. (2015)	EEG+EOG	Simultaneous	-	Vigilance estimation	Improve the performance
Kim et al. (2015)	EEG+Eye Tracker	Sequential	SVM	Curser movement	Increase the ITR
Kim et al. (2015)	EEG+Eye Tracker	Sequential	LDA	Curser movement	Increase the ITR
Lin et al. (2015)	EEG+EMG	Simultaneous	-	Speller	Increase the classification accuracy
Leeb et al. (2011)	EEG+EMG	Sequential	-	Speller	Increase the classification accuracy+ITR and the number of target items
Riccio et al. (2015)	EEG+EMG	Sequential	-	Speller	Improve the performance and ITR
Riccio et al. (2015)	EEG+EMG	Simultaneous	CCA	Speller	Increase the classification accuracy+the number of targets and ITR
Riccio et al. (2015)	EEG+EMG	Simultaneous	LDA	Object control	Increase the classification accuracy
Riccio et al. (2015)	EEG+EMG	Simultaneous	SVM	Object control	Improve the object control
Ahn et al. (2013)	EEG+SSSEP	Sequential/Simultaneous	-	Curser movement	Decrease the classification accuracy in simultaneous combination
Yao et al. (2014a)	EEG+SSSEP	Simultaneous	-	Curser movement	Providing multi-class BCI system
Yao et al. (2014b)	EEG+SSSEP	Simultaneous	LDA	Object control	Increase the classification accuracy
Yao et al. (2014a)	EEG+SSSEP	Simultaneous	LDA	Object control	Achieve optimal subjects’ performance
Khan et al. (2014a)	EEG+NIRS	Simultaneous	-	Wheelchair control	Realization a large number of commands
Yao et al. (2014b)	EEG+NIRS	Simultaneous	LDA	-	Increase the ITR
Yao et al. (2014a)	EEG+NIRS	Simultaneous	LDA	-	Provide open access dataset
Al-Shargie et al. (2016)	EEG+NIRS	Simultaneous	SVM	Stress assessment	Increase the classification accuracy and improve sensitivity and specificity

SSVEP: Steady-State Visual Evoked Potential; ERD: Event-Related Desynchronization; EEG: Electroencephalogram; EOG: Electrooculogram; EMG: Electromyogram; SSSEP: Steady-State Somatosensory Evoked Potentials; NIRS: Near-Infrared Spectroscopy; and ITR: Information Transfer Rate

#### 2.2.1. The combination of P300 and SSVEP

Since both SSVEP and P300 are evoked by visual stimulation and none of them requires training, the combination of these two signals is used in various applications such as target selection, movement control, and spellers. In order to control the direction and speed of the movement, simultaneous combination of SSVEP and P300 is associated with shortages such as low speed and ignoring the resting state in synchronous systems (Bi, Lian, Jie, Lai, & Liu, 2014).

P300, associated with high ITR, is considered the main mechanism of data transfer in many applications, including spellers. One of the solutions to increase ITR in P300-based spellers is reducing the number of flashes. There is a compromise between ITR and classification accuracy that by reducing one of them, the other one increases. In order to increase ITR, simultaneous combination of P300 and SSVEP is used. To this end, all characters are divided into subareas by two techniques: Row/Column (RC) or Subarea/Location (SL).

All of the characters in each subarea flicker at the same frequency. At the same time, cues highlight the same location in each subarea in a pseudorandom sequence. Thus, only N1 flash codes for P300 and N2 frequencies for SSVEP are required to achieve the spelling of N1×N2 items. The RC mode is a better choice compared with SL mode, because of its higher average, faster speed, and lower standard deviation of ITR (Yin et al., 2014). To increase classification accuracy, simultaneous combination of SSVEP and P300 is used to reduce errors occurred in rows or columns containing the target characters. Simultaneous with P300, several SSVEP frequencies are applied; hence, the characters in the same row or column may not have the same frequency (Yin et al., 2013). To increase classification accuracy aimed at detecting the control state (period of time in which the subject is intended to convey information), the sequential combination of P300 and SSVEP is used (Edlinger, Holzner, & Guger, 2011; Panicker, Puthusserypady, & Sun, 2011).

In simultaneous combination of P300 and SSVEP, to avoid any disruption caused by unstable frequency of P300 on the frequency of flickering SSVEP, the P300 is used as the target deformation (Wang et al., 2015). In this approach, using the stop features of SSVEP coupled with changing shape in P300 could also be effective (Xu et al., 2013).

#### 2.2.2. The combination of ERD and SSVEP

SSVEP and ERD combination is employed in various applications such as the control of wheelchair, orthotics, and neural prosthesis. In combination of these signals, the ability to use the desired control signal at any moment leads to the increase in classification accuracy (Allison et al., 2010). This combination could also be effective in increasing the number of control commands. For example, if the classification of four directions of movement is realized using the right and left wrist motor imagery and taking into account the time of imagination, it is not possible yet to control the cursor in several directions in a moment (Bai, Lin, Huang, Fei, & Floeter, 2010).

SSVEP and ERD combination enable continuous movement in two dimensions simultaneously (Allison et al., 2012). Moreover, the possibility of simultaneous control of direction and speed of the wheelchair is provided and the move/stop command is made by spending a short time (Li et al., 2013; Cao, Li, Ji, & Jiang, 2014; Li et al., 2014). To increase the ITR, the combination of these two signals is practical and better than switching from one state to another, since the fatigue resulting from this approach is not much (Brunner, Allison, Altstätter, & Neuper, 2011).

To increase classification accuracy, their combination is used to detect resting state in various applications such as opening/closing orthotics and controlling neural prosthetics during grasping. The division of task into two steps and the possibility to turn off the LED after the completion of the first step reduce the fatigue and error rate by reduction of the adverse impact of LED flashes on ERD detection (Pfurtscheller, Solis-Escalante, Ortner, Linortner, & Muller-Putz, 2010; Savić, Kisić, & Popović, 2011).

#### 2.2.3. The combination of P300 and ERD

The most common practical applications of P300 and ERD combination are wheelchair and robot control. In general, the control of these objects is done in two ways. In the first case, several targets are shown against the subject and the subject should select one of them. The subject automatically moves towards the target through a predetermined path. In this case, the individual has no control over the path. In the second case, the subject moves himself closer to the target with the voluntary movements in different directions. In order to automatically move the wheelchair, sequential combination of P300 and ERD is used.

In the first stage, the target is selected using P300 and the subject moves himself closer to it from a predetermined path. In the second stage, the ERD is used for stop command (Rebsamen et al., 2008; Rebsamen et al., 2010; Riechmann, Hachmeister, Ritter, & Finke, 2011). On the other hand, when the voluntary control of direction and the speed of movement are considered, both simultaneous and sequential combinations are used.

Typically, in applications that employ the simultaneous combination of these two control signals, ERD is used to move in different directions and P300 is applied to control the speed or stop command (Long et al., 2012a). In sequential combination, ERD is used for routing and P300 is applied to achieve the desired object. Utilization of ERD for routing limits the number of commands and the P300 provides the control panel for the subject that allows him the possibility of further tasks (Finke, Knoblauch, Koesling, & Ritter, 2011; Su et al., 2011).

#### 2.2.4. The combination of EEG and EOG

EEG-based systems are a superior technology to increase the communication of patients with disability and or paralysis that cannot move and speak. However, if there is little ability to move eyes in patients, this ability can also be used in conjunction with EEG signals in HBCI systems. Eye movement changes the orientation of the corneal-retinal potential and the electrodes placed around the eyes can record the effects named EOG. The combination of EOG signal and other control signals is used in various applications such as control of virtual keyboard, wheelchair, mobile robot, etc.

The input of eye movement does not require much training and acts very fast. The amplitude of EOG signal is about several microvolts, hence, it could be easily classified with high accuracy. This method is economically affordable since the number of electrodes is few. As an example of this combination in robot control, moving to the right and left direction is obtained using only two EOG electrodes. Direct movement and complete stop are also done by motor imagery and eye closing, respectively (Usakli, Gurkan, Aloise, Vecchiato, & Babiloni. 2009; Punsawad, Wongsawat, & Parnichkun, 2010).

#### 2.2.5. The combination of EEG and Eye-tracker

Eye tracking system is a wearable human-computer interface that provides the possibility to communicate through eye movements and blinking. The combination of this interface and EEG signals could be used in HBCI systems. The main use of this combination is curser movement on the screen. First, the subject guides the curser to the target as quickly as possible and then selects it. Eye motion indicates the cursor movement on the screen and the target is selected by EEG signal. Although the ITR in this combination is less than using mouse, this rate is increased compared with that of BCI (Kim, Kim & Jo, 2015).

#### 2.2.6. The combination of EEG and EMG

Some patients may have little ability to move muscles in some organs. In many applications, this residual motion is not useful to control objects due to muscle weakness, exhaustion or disruption of natural tension. However, this ability can be effectively employed as a second signal in HBCI systems. For each patient, the suitable muscle is selected for electrode placement based on its ability to contract (Lalitharatne, Teramoto, Hayashi, & Kiguchi, 2013). The combination of EMG and motor imagery, P300 and SSVEP, is employed in various applications. SSVEP-based speller despite high ITR, high signal to noise ratio, and no need for training, only has appropriate response in a certain frequency range; it limits the number of target items.

To increase ITR and the number of characters in spellers, sequential combination of SSVEP and EMG is used, in such a way that all characters are divided into several groups and the ones that are in the same group flicker with different frequencies. The number of muscle activities determines the group number. Hence, after determining the desired group, the target item is selected by SSVEP (Lin, Chen, Huang, Ding, & Gao, 2015). To increase classification accuracy, the simultaneous combination of motor imagery and EMG has relatively better results in comparison with that of BCI system (Leeb, Sagha, Chavarriaga, & del R Millán, 2011). In this regard, P300 and EMG combination can be used to correct the error in spellers. In other words, contrary to BCI systems, which use backspace to delete the wrong letter, it is realized with the EMG in hybrid mode (Riccio et al., 2015).

#### 2.2.7. The combination of EEG and SSSEP

Many people with stroke that their muscles are damaged as well as people who lost the ability of eye gaze may have the ability to feel stimulation, which can be used in HBCI systems. For example, a combination of steady state somatosensory evoked potential retrieved from selective sensation and motor imagery is used in cursor control. Motor imagery is the activation of efferent motor nerves and selective sensation is receiving afferent neuron inputs related to stimulation perception. ERD and SSSEP are achieved by motor imagery and tactile stimulation, respectively. In the simultaneous combination of these signals, increasing the classification accuracy could not be achieved due to ERD degradation caused by tactile signals (Ahn, Ahn, Cho, & Jun, 2013). In fact, ERD reduces the SSSEP amplitude and selective sensation increases it (Yao, Meng, Zhang, Sheng, & Zhu, 2014a).

#### 2.2.8. The combination of EEG and NIRS

Non-invasive brain imaging technique that uses light with a wavelength range of 600 to 1000 nm is called NIRS. It is used to measure the hemodynamic response due to oxygenated hemoglobin, hemoglobin without oxygen, water, etc. EEG signal has good temporal resolution but poor spatial resolution, while NIRS has moderate temporal and spatial resolution and is also resistant to noise (Coyle, Ward, Markham, & McDarby, 2004; Herff et al. 2015). In HBCI systems, EEG, and NIRS combination is used to increase the number of commands without reduction in classification accuracy. For example, NIRS is used to measure brain activity caused by mental acts (mental counting or performing subtraction) and EEG signal is applied to detect movement (Khan, Hong, Naseer, Bhutta, & Yoon, 2014a).

## 3. Discussion

The current study assessed different HBCI systems that were the result of the combination of a BCI control signal and other BCI control signals or HMI control biosignals from the perspective of application, capabilities, and limitations. HBCI systems are used in many applications such as object control, movement control, spellers, etc. By the sequential combination of these systems, a complex task can be divided into several stages and only one BCI system is used at each stage. This method of combination assists to reduce the errors by better distinguishing the rest state from the attention state. On the other hand, the main goal of simultaneous combination of these systems is to increase the ITR. Generally, HBCI systems have higher ITR and greater classification accuracy compared to those of the conventional BCI systems, but they are usually more complex. This complexity can affect the ease of use of the system and its acceptance by the user.

**Figure 5.**
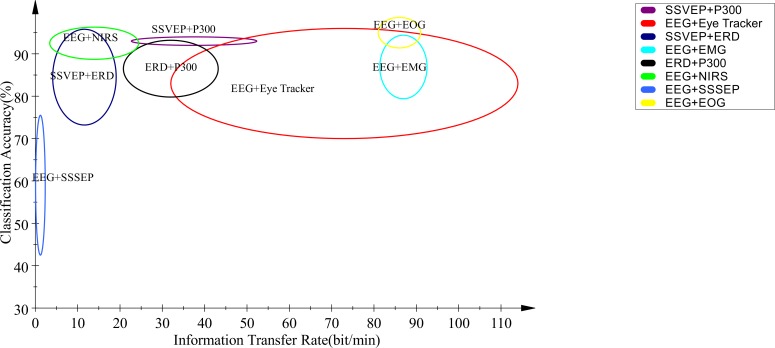
The comparison of different combinations of HBCI systems based on classification accuracy and information transfer rates The range of classification accuracy and ITR were determined based on the conducted researches.

From this perspective, the design and implementation of these systems including the number of channels play an important role in the performance of system. Table 2 summarizes results of different studies with an emphasis on the number of channels, ITR, and classification accuracy. The experimental conditions and signal recording considerations are different in these studies. Accuracy and ITR measures are sensitive to the experiment protocol, which makes it difficult to compare the results. Hence, to manage this issue, these measures are presented in the graphical form of Figure 5. In this Figure, various HBCI combinations were compared with two quantitative criteria of classification accuracy and ITR. In this Figure, an ellipse is drawn for the ranges of ITR and classification accuracy of each method.

**Table 2.** A comparison of the results of various researches in the field of hybrid BCI

**Paper**	**Combined Control Signal**	**Combination Type**	**The Number of Channel**	**Classification Accuracy**	**Information Transfer Rate**
Panicker et al. (2011)	P300+SSVEP	Sequential	9	88.15	19
Xu et al. (2013)	P300+SSVEP	Sequential	32	97.5±6.2	34
Yin et al. (2013)	P300+SSVEP	Simultaneous	12	93.85±49.7	56.44±8.19
Yin et al. (2014)	P300+SSVEP	Simultaneous	12	95±5	48±4
Wang et al. (2015)	P300+SSVEP	Simultaneous	8	90.63±10.16	22±52.6
Wang et al. (2015)	P300+SSVEP	Sequential	3	93±1	-
Wang et al. (2015)	P300+SSVEP	Sequential	12	94±6	-
Wang et al. (2015)	P300+SSVEP	Simultaneous	14	93±7	32±5.5
Allison et al. (2010)	SSVEP+ERD	Simultaneous	6	81±8.9	-
Brunner et al. (2011)	SSVEP+ERD	Simultaneous	8	95.6±6.7	4.7±2.4
Pfurtscheller et al. (2010)	SSVEP+ERD	Sequential	2	85±6	-
Savić et al. (2011)	SSVEP+ERD	Sequential	6	98	-
Allison et al. (2012)	SSVEP+ERD	Simultaneous	35	60	-
Cao et al. (2014)	SSVEP+ERD	Simultaneous	15	90±2	-
Wang et al. (2015)	SSVEP+ERD	Sequential	13	11±77	
Wang et al. (2015)	SSVEP+ERD	Simultaneous	15	89±5.5	
Wang et al. (2015)	SSVEP+ERD	Simultaneous	14	91±1	1±17
Bhattacharyya et al. (2014)	P300+ERD	Sequential	19	95	1±22.5
Long+et al. (2012b)	P300+ERD	Sequential	30	93.99	-
Roula et al. (2012)	P300+ERD	Sequential	14	12.7±85	-
Riechmann et al. (2011)	P300+ERD	Simultaneous	16	79.5±2.5	-
Riechmann et al. (2011)	P300+ERD	Sequential	18	88±4	11±41
Riechmann et al. (2011)	P300+ERD	Sequential	18	87±4	10±43
Punsawad et al. (2010)	EEG+EOG	Simultaneous	5	96±2	-
Ramli et al. (2015)	EEG+EOG	Sequential	5	97.88	86±5
Ma et al. (2015)	EEG+EOG	Sequential	10	11.5±87	-
Koo et al. (2014a)	EEG+EOG	Sequential	10	80	56
Riechmann+et al. (2011)	EEG+EOG	Simultaneous	15	1.7±91	-
Meena et al. (2015)	EEG+Eye Tracker	Simultaneous	2	5±60	-
Kim et al. (2015)	EEG+Eye Tracker	Sequential	14	95.6±2.6	-
Kim et al. (2015)	EEG+Eye Tracker	Sequential	14	84±9.5	120±7.5
Dong et al. (2015)	EEG+Eye Tracker	Simultaneous	32	>80	-
Riechmann et al. (2011)	EEG+Eye Tracker	Sequential	16	85.5±2	
Riechmann et al. (2011)	EEG+Eye Tracker	Sequential	-	95.3±1.5	40.5±1
Riechmann et al. (2011)	EEG+Eye Tracker	Sequential	4	79	60.4
Lin et al. (2015)	EEG+EMG	Sequential	11	80.8±15.6	83.7±24
Leeb et al. (2011)	EEG+EMG	Simultaneous	20	91	-
Riccio et al. (2015)	EEG+EMG	Sequential	9	100	12
Leeb et al. (2010)	EEG+EMG	Simultaneous	16	88	-
Kiguchi et al. (2012)	EEG+EMG	Simultaneous	16	84±7.7	-
Riechmann et al. (2011)	EEG+EMG	Simultaneous	11	86±9	91±16
Ahn et al. (2013)	SSSEP+EEG	Sequential/Simultaneous	64	64±5.5	-
Ahn et al. (2013)	SSSEP+EEG	Simultaneous	64	77	1.2±1.14
Yao et al. (2014a)	SSSEP+EEG	Simultaneous	64	83±8.5	-
Riechmann et al. (2011)	SSSEP+EEG	Simultaneous	32	44.5±7	-
Riechmann et al. (2011)	SSSEP+EEG	Simultaneous	32	55.5±8.5	-
Khan et al. (2014a)	EEG+NIRS	Simultaneous	20	>80	-
Fazli et al. (2012)	EEG+NIRS	Simultaneous	37	86±5	-
Koo et al. (2015b)	EEG+NIRS	Simultaneous	8	88±10	-
Khan et al. (2014b)	EEG+NIRS	Sequential	3	80	-
Lee et al. (2014)	EEG+NIRS	Simultaneous	37	59	-
Ma et al. (2012)	EEG+NIRS	Simultaneous	2	83	-
Herff et al. (2015)	EEG+NIRS	Simultaneous	3	58±14.5	-
Tomita et al. (2014)	EEG+NIRS	Simultaneous	2	-	13.9±10.5
Riechmann et al. (2011)	EEG+NIRS	Simultaneous	23	94±3.5	-
Riechmann et al. (2011)	EEG+NIRS	Simultaneous	31	95±4	-

The center of ellipse and its diameters are set based on the mean and standard deviation of average values listed in Table 2. Classification accuracy and ITR are two important parameters to evaluate a BCI system. It is a tradeoff between these two parameters; as one increases, the other one decreases and vice versa. For a particular application, the increase in accuracy may be considered an advantage or an increase in ITR is desirable. Therefore, concerning the ultimate goal and depending on the application, the appropriate combination type should be determined. By having the correct location of the accuracy and the ITR corresponding to each combination, it is easy to determine the optimal combination.

The current study results showed that in most cases, the combination of a BCI control signal and HMI control biosignal had relatively higher ITR in comparison with those of the combination of the two BCI control signals. According to the Figure, the highest and lowest ITRs were achieved using EEG and Eye Tracker and SSVEP and ERD, respectively. The combination of EEG signal and NIRS also had the lowest classification accuracy in comparison with those of the others, while the accuracy values of other hybrid systems did not differ much from each other and they were located within the range of 70% to 100%. Generally, in using HBCI systems, the combination technique can be determined based on the type of application, the main goal, as well as the capabilities of patients.

## Ethical Considerations

### Compliance with ethical guidelines

There is no ethical principle to be considered doing this research.
